# Single-cell transcriptome sequencing revealing the difference in photosynthesis and carbohydrate metabolism between epidermal cells and non-epidermal cells of *Gracilariopsis lemaneiformis* (Rhodophyta)

**DOI:** 10.3389/fpls.2022.968158

**Published:** 2022-11-17

**Authors:** Haihong Chen, Yiyi Hu, Pingping Li, Xiaoqing Feng, Min Jiang, Zhenghong Sui

**Affiliations:** Key Laboratory of Marine Genetics and Breeding, Ministry of Education, Ocean University of China, Qingdao, China

**Keywords:** photosynthesis, carbohydrate metabolism, photoassimilates allocation, floridioside, floridean starch, agar, *Gracilariopsis lemaneiformis*, single-cell transcriptome sequencing

## Abstract

The allocation of photoassimilates is considered as a key factor for determining plant productivity. The difference in photosynthesis and carbohydrate metabolism between source and sink cells provide the driven force for photoassimilates’ allocation. However, photosynthesis and carbohydrate metabolism of different cells and the carbon allocation between these cells have not been elucidated in *Gracilariopsis lemaneiformis*. In the present study, transcriptome analysis of epidermal cells (EC) and non-epidermal cells (NEC) of *G. lemaneiformis* under normal light conditions was carried out. There were 3436 differentially expressed genes (DEGs) identified, and most of these DEGs were related to photosynthesis and metabolism. Based on a comprehensive analysis both at physiological and transcriptional level, the activity of photosynthesis and carbohydrate metabolism of EC and NEC were revealed. Photosynthesis activity and the synthesis activity of many low molecular weight carbohydrates (floridoside, sucrose, and others) in EC were significantly higher than those in NEC. However, the main carbon sink, floridean starch and agar, had higher levels in NEC. Moreover, the DEGs related to transportation of photoassimilates were found in this study. These results suggested that photoassimilates of EC could be transported to NEC. This study will contribute to our understanding of the source and sink relationship between the cells in *G. lemaneiformis*.

## Highlights

Single-cell transcriptome sequencing revealed the difference in photosynthesis as well as carbohydrate metabolism between EC and NEC of *Gracilariopsis lemaneiformis*, and photoassimilates might be transported from EC to NEC.

## Introduction

In plants, photoassimilates are produced in source tissues (photosynthetically active tissues, such as leaves) and then allowed to be transported to support the growth of sink tissues such as fruits or roots which themselves are unable to produce assimilates or the assimilates produced by themselves are not enough to support their own needs (Hofius and Börnke, 2007; Albacete et al., 2014; Suh et al., 2021).

Past researches have greatly advanced our understanding of the allocation of photoassimilates in higher plants compared with red algae. In vascular plants, photoassimilates produced by mesophyll cells are usually loaded into the phloem at first, and then transported long distance and unloaded into the distal sink parenchyma cells (Hofius and Börnke, 2007; Osorio et al., 2014). However, the unique living environment and relatively simple structure of red algae might lead to great differences in carbohydrate metabolism and photoassimilates partitioning compared with higher plants. For example, sucrose is the main low molecular weight carbohydrates (LMWC) for respiration and biosynthesis in most higher plants and it is also the main form of photoassimilates transferred between source and sink cells in these plants, while there was extremely low concentration of sucrose in red algae (Lemoine et al., 2013; Lv et al., 2019). Currently, photoassimilates transporting and partitioning between red algae cells have been poorly investigated yet.

Previous studies have shown that photosynthesis and carbohydrate metabolism of cells have a vast influence on both plant growth and development since their differences between cells provide the driven force for carbon transporting and partitioning, thus keeping carbon balance in different cells/tissues (Hofius and Börnke, 2007; Osorio et al., 2014). Evidence has shown that changes in the activities of photosynthesis and carbohydrate metabolism affected the photoassimilates’ allocation and the growth of plant (Yu et al., 2004; Hofius and Börnke, 2007; Rosa et al., 2017). Therefore, a better understanding of the difference in photosynthesis and carbohydrates metabolism between cells is important for revealing the photoassimilates’ allocation between these cells and for further investigating the regulation mechanism of the growth and development of economic species.

**Figure 1:**
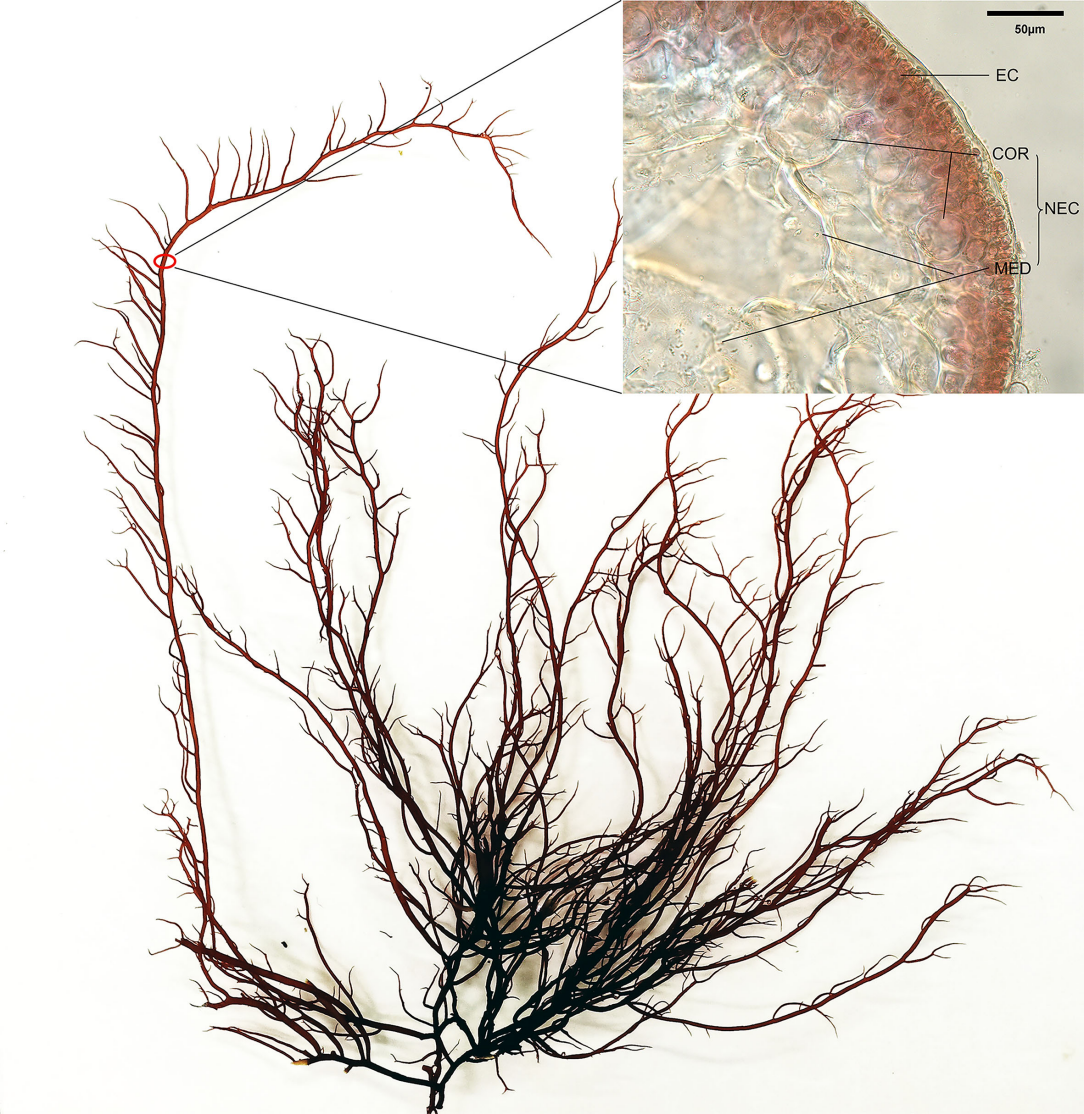
Alga and the cells in the branch of *G. lemaneiformis*, EC, epidermal cell; NEC, non-epidermal cell; COR, cortical cell; MED, medullary cell.

*Gracilariopsis lemaneiformis* is an important economical seaweed belonging to Rhodophyta, Florideophyceae, and Gracilariaceae. It has attracted increasing attention owing to its excellent agar yield and quality (González-Leija et al., 2009), as well as high economic value in aquaculture, food production, and eutrophication seawater treatment (Pan et al., 2016; Yu et al., 2016; Wei et al., 2017). The alga in the non-reproductive period is mainly composed of epidermal cells (EC) and non-epidermal cells (NEC, including cortical cells (COR) and medullary cells (MED)) (Chen et al., 2020). Among them, ECs locate on the surface of the alga about 2~3 layers with high pigment content. CORs locate between EC and MED, and the pigment content is lower than that of EC. MEDs locate in the center of the algal branch and the pigment is barely visible (**Figure 1**). The large differences in the pigment content and light environment suggested that the capacity and activity of photosynthetic carbon fixation of EC and NEC is different, which might result in allocation of photoassimilates between these cells.

In the present study, single cell transcriptomic sequence technology was performed to reveal the differences in photosynthesis and metabolism between EC and NEC of non-reproductive *G. lemaneiformis* under normal light conditions. Expression of the genes related to the photosynthesis, the main carbohydrate metabolism, and the photoassimilates’ transportation in EC and NEC were analyzed. Chlorophyll fluorescence of the cells as well as some carbohydrates of EC and NEC were measured in this study. Furthermore, the allocation of carbohydrates between EC and NEC were discussed.

## Materials and methods

### Materials and growth conditions

*G. lemaneiformis* were collected from aquaculture areas of Rongcheng, Shandong Province, China. Then, shoot apices of about 1 cm were cultured in Provasoli’s medium (Stein, 1973) at 20°C in a 12h/12h light/dark cycle and illumination intensity of 30 μmol photons m^−2^s^−1^ for a month before the experiment.

### Single cell isolation and collection

*G. lemaneiformis* with approximately 3-4 cm shoot apices were collected, and the epidermal and non-epidermal tissues were isolated using a scalpel under an anatomic microscope. Then, the tissues were incubated in mixed enzymes solution (2% w/v cellulase, 1% w/v macerozyme R-10, 0.8 M sorbitol, 600 μL of crude *Marinomonas* sp. YS-70 agarase solution, and 400 μL of deionized water) for 1 h at 27°C (Chen et al., 2018). After that, the ECs were isolated from the epidermal tissues using a 10 μm filter screen and collected from the filtrate following centrifugation (200 ×g for 8 min). The NECs were obtained from the enzyme solution containing non-epidermal tissues by centrifugation at 200×g for 8 min. Both ECs and NECs were washed with deionized water containing 1.5 M sorbitol and collected by centrifugation.

### Preparation of cDNA and RNA-Seq library construction and sequencing

Approximately 1 μL of single-cell suspension of EC and NEC (containing approximately 100 ECs or 50 NECs) was transferred respectively to a 0.2 mL PCR tube, mixed with 2 µL of lysis buffer (Fan et al., 2021), and stored at −80°C until further use. The cDNA amplification, library preparation, and sequencing were performed by Annoroad Gene Tech. (Beijing) Co., Ltd. The amplified cDNA was prepared using SMARTer Ultra Low RNA Kit (Clontech, Mountain View, CA, USA), and RNA-Seq library was constructed using NEBNext Ultra DNA Library Prep Kit for Illumina (NEB, Ipswich, MA, USA). The constructed libraries were sequenced using Illumina Novaseq platform with sequencing strategy PE150 to obtain 150 bp paired-end reads.

### Transcriptome data processing and differentially expressed genes functional analysis

By trimming Smart-seq2 public primer sequences from the raw reads and removing low-quality reads, adapters, and unknown nucleotides (N> 5%) using Perl, clean reads (high-quality reads) were obtained. Then, Bowtie2 was used for building the genome index (Langmead & Salzberg, 2012), and clean reads were subsequently aligned to the reference genome using HISAT2 (Kim et al., 2019). The reads count for each gene in each sample was obtained using HTSeq (Anders et al., 2014), and gene expression levels were estimated using FPKM (fragments per kilobase million mapped reads) (Wagner et al., 2012). Finally, DEGs analysis was performed using DESeq2 (Wang et al., 2009). Genes with q value ≤ 0.05 and |log_2_(fold change)|≥1 were identified as DEGs, where q value denotes the correct form of p value and Log_2_(Fold Change) indicates the logarithmic form of the fold change value to base 2.

Uni-transcripts were functionally annotated at a series of databases, including the non-redundant protein database (Nr, ftp://ftp.ncbi.nih.gov/blast/db/), the Kyoto Encyclopedia of Genes and Genomes (KEGG, http://www.genome.jp/kegg/), and gene ontology (GO, http://www.geneontology.org/). GO and KEGG function enrichment were performed using GO and KEGG enrichment analysis in OmicShare, an online platform for data analysis (www.omicshare.com/tools).

### Quantitative real-time PCR analysis

RNA was extracted from 0.05 g of each sample (EC and NEC tissues) using an E.Z.N.A. Plant RNA Kit (Omega Bio-tek Inc., Norcross, GA, USA) following the manufacturer’s instructions. Genomic DNA was removed and first strand cDNA was synthesized using the RT Reagent Kit (Takara, Dalian, China) following the manufacturer’s instructions. The cDNA was stored at -80°C until use. All samples were analyzed in triplicate.

Ubiquitin-conjugating enzyme (*UBC*), a widely used reference gene whose expression was not significant in EC and NEC, was selected for real-time PCR normalization (Niu et al., 2015). The primers for genes are shown in **Table S1**. All qRT-PCR analyses were performed using a 7500 Fast Real-time PCR System (Applied Biosystems, Carlsbad, CA, USA). One μL of cDNA, 10 μL of SYBR Green Real-time PCR Master Mix (including MgCl_2_, dNTPs, Taq DNA polymerase, SYBR Green I) (Roche, Basel, Switzerland), 0.8 μL of each respective primer, and 7.4 μL of molecular grade H_2_O comprise the 20 μL reaction mixture. PCR amplification was performed as follows: initial denaturation at 95°C for 1 min followed by 40 cycles of 95°C for 15 s and 60°C for 1 min. The qRT-PCR results were analyzed using the 2^−ΔΔCt^ method (Livak & Schmittgen, 2001).

### Detection of soluble sugars in the cells of *G. lemaneiformis*

EC and NEC tissues were freeze-dried and pulverized. The pulverized ECs and NECs tissues (7.5mg) were added in 700 μL 80% ethanol respectively and shocked at 50 °C for 2h. Then 700 μL H_2_O were added in each sample and the samples were centrifuged with 10000 rpm for 3 min. The supernatant was used for chromatographic detection. Each type of sugar (100mg) from **Table S2** were added in 50% methanol to make a standard sample of 20 μg/mL for chromatographic detection. The chromatography system used Thermo ICS5000 (Dionex, Thermo Scientific, Waltham, US) ion chromatography system with a CarboPac ™ PA20 (3 x 150mm) liquid chromatography column. The flow phase was A: H_2_O and B:100 mM NaOH, the sample was 5uL, the flow rate was 0.5 mL/min, and the column temperature was 30°C. Elution gradient: 0min A/B (95:5V/V), 9min A/B (95:5V/V), 20min A/B (0:100 V/V), 30min A/B (0:100 V/V), 30.1min A/B (95:5 V/V), 40min A/B (95:5 V/V). Then, the data was obtained and processed using Chromeleon 6.8 (Chromatography Data System Software).

### Measurement of chlorophyll fluorescence parameters

The chlorophyll fluorescence parameters were measured using MicrofluorCam (Photon Systems Instruments, Czech Republic). The program was set on MicrofluorCam→Protocol. After 20 min dark adaptation, the chlorophyll fluorescence parameters were captured by the apparatus, including dark-adapted minimum fluorescence (F_0_), dark-adapted maximum fluorescence (F_m_), light-adapted maximum fluorescence (F_m_’), light-adapted minimum fluorescence (F_0_’), and light-adapted real-time fluorescence yield (F). Then, the maximum photosynthetic efficiency (F_v_/F_m_) (Kitajima & Butler, 1975), the photochemical quenching (qL) (Kramer et al., 2004), and the actual photosynthesis (Y(II)) (Genty et al., 1989) were determined as follows:


$$\frac{F_{v}}{F_{m}}=\left(F_{m}-F_{0}\right)/F_{m}$$

$$qL=\left(F_{m}^{'}-F\right)/\left(F_{m}^{'}-F_{0}^{'}\right)*F_{0}^{'}/F$$

$$Y\left(\mathrm{II}\right)=\left(F_{m}^{'}-F\right)/F_{m}^{'}$$

### Electron microscopy

The 3 mm shoot apices were harvested and fixed overnight in 2.5% glutaraldehyde at 4°C. Then, the samples were fixed in 1% osmium tetroxide. After fixation in osmium tetroxide, the samples were dehydrated with ethanol treatments (50%, 70%, 80%, 90%, 95%, and 100%; 10 min for each gradient). Subsequently, these samples were embedded in Epon 812 and cured at 37°C, 45°C, and 65°C, 24 h for each temperature (Dos Santos et al., 2013). After that, the samples were cut with a Reichert-Jung Ultracut E Ultramicrotome (Germany) and a JEOL JEM-1230 transmission electron microscope (Japan) was used to examine the thin sections stained with saturated uranyl acetate and lead citrate.

### Statistical analysis

R and SPSS were used to analyze the obtained data. In comparisons between two groups, Student’s t-test was used, or analysis of variance (ANOVA) followed by one-way ANOVA for comparisons between more than two groups.

## Results

### Transcriptome sequencing and gene expression of EC and NEC

**Figure 2:**
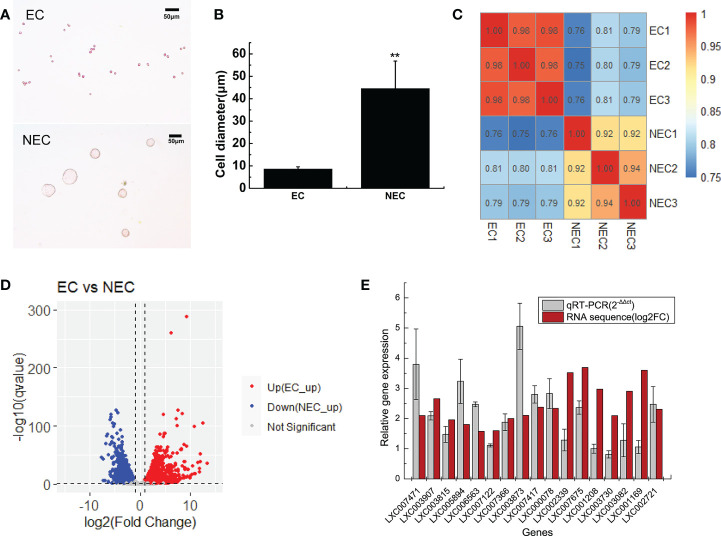
Transcriptome analyses of ECs and NECs of *G. lemaneiformis*. **(A)** Single cells of ECs and NECs. **(B)** Diameter of ECs and NECs. “**” indicated statistically significant differences by Student’s t-test (p < 0.01). **(C)** Correlation plot of gene expression levels in ECs and NECs. The numbers in boxes represented Spearman correlation coefficient of the corresponding two samples. **(D)** Volcano plot of the DEGs levels in ECs and NECs. **(E)** Validation of gene expression of some DEGs by qRT-PCR. Log2(fold change) represents logarithmic value of the change in expression of NEC vs EC.

In this study, single-cell transcriptome sequencing analyses were conducted on isolated ECs (8.5 ± 1 μm) and NECs (44.5 ± 12.3 μm) of *G. lemaneiformis* (**Figures 2A, B**). After filtering the sequencing data, a total of 53.92 G of clean data and 361,279,376 clean reads were obtained from the two groups of cells. Among them, 95.38% and 95.47% of the clean reads of EC and NEC had Phred-like quality scores at the Q30 threshold. Of the clean reads, 95.95% and 90.11% of EC and NEC were aligned to the genome, respectively (**Table 1**). Spearman correlation coefficient of gene expression for the same cell group was >0.9, while that for different cell groups was<0.81, indicating gene expression profiles of ECs and NECs were different (**Figure 2C**). There were 3436 DEGs screened using thresholds of |log2 (fold change)| ≥1 and q value ≤ 0.05, among which 1785 and 1651 DEGs were upregulated in ECs and NECs, respectively (**Figure 2D**). To further evaluate the quality of the transcriptome, we examined the expression patterns of 17 genes that were significantly up regulated in NEC. Results showed that the gene expression patterns of RNA-seq data were mostly consistent with qRT-PCR data (**Figure 2E**). These results indicated that RNA-seq data of this study were trustworthy and appropriate for further analysis.

**Table 1:** Transcriptome sequencing results for ECs and NECs of *G. lemaneiformis*.

Sample	Clean Bases Number (G)	Q30	Clean Reads	Clean Reads rate(%)	Mapping rate (%)	Total gene number
EC1	9.06	95.47	60822770	92.58	96.20	7618
EC2	7.91	95.39	53153776	87.74	95.69	7672
EC3	7.45	95.28	49890170	92.52	95.97	7509
Average	8.14	95.38	54622238	90.95	95.95	7600
NEC1	9.11	95.56	60981670	95.09	86.37	7220
NEC2	10.13	95.49	67779426	94.69	91.51	7067
NEC3	10.26	95.38	68651564	93.76	92.46	7038
Average	9.83	95.47	65804220	94.51	90.11	7108

### Function annotation of DEGs of EC vs NEC

**Figure 3:**
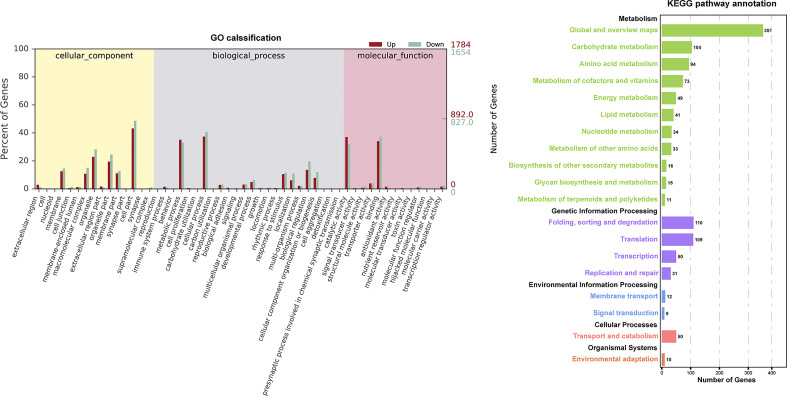
Histogram of Gene Ontology (GO) classifications and Kyoto Encyclopedia of Genes and Genomes (KEGG) pathways annotation for the differentially expressed genes (DEGs) of EC and NEC. Up indicated genes up-regulated in EC. Down indicated genes up-regulated in NEC.

The GO and KEGG database were used to classify the function of DEGs. The DEGs were categorized into ‘cellular component’ ‘biological process’ and ‘molecular function’(**Figure 3**). In the “cellular component” category, many DEGs were annotated as “cell part”, “organelle”, and “organelle part”. The “cellular process” and “metabolic process” were the most abundant subcategories within the “biological process”, while “catalytic activity” and “binding” were the major groups within the “molecular function”. The DEGs of EC vs NEC were found to be significantly enriched in 12 GO terms (q<0.05), among which most of the terms were related to photosynthesis, such as “thylakoid part”, “photosynthetic membrane”, and “thylakoid membrane” (**Table 2**). Moreover, “cytoplasmic, membrane-bounded vesicle” were also significantly enriched by DEGs (**Table 2**).

**Table 2:** Gene Ontology (GO) significantly enriched terms for differentially expressed genes (DEGs) of EC vs NEC.

ID	Term	Class	Ratio^1^	pvalue	qvalue
GO:0044436	thylakoid part	Cellular Component	0.75	2.59E-08	2.14E-05
GO:0034357	photosynthetic membrane	Cellular Component	0.76	8.65E-08	2.14E-05
GO:0042651	thylakoid membrane	Cellular Component	0.76	8.65E-08	2.14E-05
GO:0009579	thylakoid	Cellular Component	0.75	1.01E-07	2.14E-05
GO:0055035	plastid thylakoid membrane	Cellular Component	0.74	1.94E-05	0.003308
GO:0009535	chloroplast thylakoid membrane	Cellular Component	0.74	0.000032	0.004548
GO:0015979	photosynthesis	Biological Process	0.77	1.08E-06	0.006112
GO:0004601	peroxidase activity	Molecular Function	0.77	2.24E-05	0.019227
GO:0016684	oxidoreductase activity, acting on peroxide as acceptor	Molecular Function	0.77	2.24E-05	0.019227
GO:0031976	plastid thylakoid	Cellular Component	0.67	0.000161	0.019623
GO:0009534	chloroplast thylakoid	Cellular Component	0.67	0.000242	0.025754
GO:0016023	cytoplasmic, membrane-bounded vesicle	Cellular Component	0.62	0.000331	0.031355

^1^Ratio presents the ratio of the number of DEGs to the number of all annotated genes in the pathway.

KEGG annotation showed that DEGs were widely involved in metabolism, especially in the “Global and overview maps” and “Carbohydrate metabolism” (**Figure 3**), indicating that metabolism of EC and NEC had been largely differentiated. In the top 20 KEGG enrichment pathway, 85% of pathways belonged to “Metabolism” (KEGG A class), including “Porphyrin and chlorophyll”, “Photosynthesis - antenna proteins”, and “Photosynthesis” pathways (**Table 3**). In addition, Cellular Process pathway related to vesicle transportation was enriched by DEGs, such as endocytosis (**Table 3**).

**Table 3:** Top 20 Kyoto Encyclopedia of Genes and Genomes (KEGG) pathways enriched by differentially expressed genes (DEGs) of EC vs NEC.

KEGG_A_class	KEGG_B_class	Pathway	Ratio^1^	pvalue	qvalue	Pathway ID
Genetic Information Processing	Folding, sorting and degradation	Protein processing in endoplasmic reticulum	0.63	0.001931	0.145367	ko04141
Metabolism	Metabolism of cofactors and vitamins	Porphyrin and chlorophyll metabolism	0.74	0.002528	0.145367	ko00860
Metabolism	Global and overview maps	Biosynthesis of secondary metabolites	0.52	0.004746	0.181937	ko01110
Metabolism	Global and overview maps	2-Oxocarboxylic acid metabolism	0.69	0.012962	0.37266	ko01210
Metabolism	Energy metabolism	Photosynthesis - antenna proteins	1	0.019973	0.401446	ko00196
Metabolism	Metabolism of cofactors and vitamins	Nicotinate and nicotinamide metabolism	0.72	0.020945	0.401446	ko00760
Metabolism	Energy metabolism	Nitrogen metabolism	0.8	0.030336	0.447196	ko00910
Metabolism	Global and overview maps	Metabolic pathways	0.48	0.031109	0.447196	ko01100
Metabolism	Amino acid metabolism	Arginine biosynthesis	0.67	0.043261	0.552779	ko00220
Genetic Information Processing	Folding, sorting and degradation	Protein export	0.65	0.065573	0.690358	ko03060
Metabolism	Energy metabolism	Photosynthesis	0.83	0.074288	0.690358	ko00195
Metabolism	Global and overview maps	Biosynthesis of amino acids	0.53	0.076311	0.690358	ko01230
Metabolism	Metabolism of other amino acids	Glutathione metabolism	0.61	0.079674	0.690358	ko00480
Metabolism	Carbohydrate metabolism	Amino sugar and nucleotide sugar metabolism	0.59	0.084044	0.690358	ko00520
Metabolism	Metabolism of other amino acids	Selenocompound metabolism	0.63	0.136555	0.860464	ko00450
Metabolism	Amino acid metabolism	Alanine, aspartate and glutamate metabolism	0.58	0.149977	0.860464	ko00250
Cellular Processes	Transport and catabolism	Endocytosis	0.55	0.152927	0.860464	ko04144
Metabolism	Carbohydrate metabolism	Pyruvate metabolism	0.56	0.153755	0.860464	ko00620
Metabolism	Carbohydrate metabolism	Ascorbate and aldarate metabolism	0.64	0.187125	0.860464	ko00053
Metabolism	Amino acid metabolism	Lysine biosynthesis	0.62	0.193428	0.860464	ko00300

^1^Ratio presents the ratio of the number of DEGs to the number of all annotated genes in the pathway.

### Different regulation of the genes involved in photosynthesis between EC and NEC

The photosynthesis process of red algae was divided into the light-reaction and dark-reaction stage, both of which take place in the plastids (Su et al., 2010; Moriyama et al., 2014). In the present study, the DEGs of EC vs NEC enrichment results showed that many GO terms were related to photosynthetic organelles such as “plastids”, “photosynthetic membrane”, and “thylakoid” (**Table 2**). Therefore, in the current study, the genes related to light and dark reaction were searched based on synonyms in the combined functional annotation and standard gene names, and their expression pattern in EC and NEC were analyzed.

**Figure 4:**
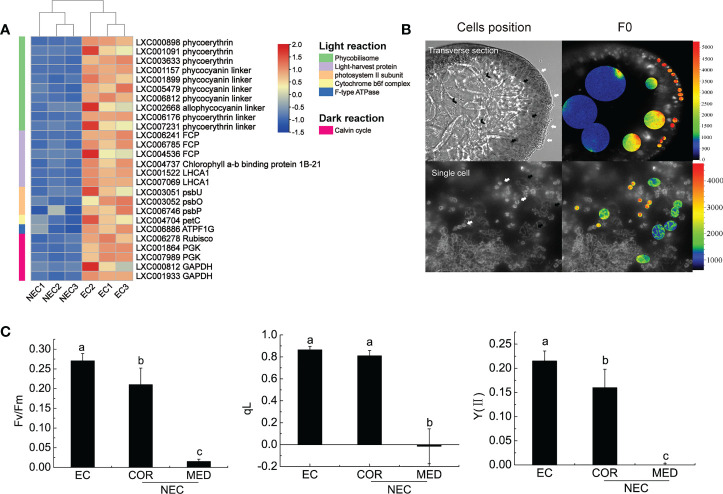
Photosynthesis of EC and NEC. **(A)** Heat map displaying the expression levels of differentially expressed genes (DEGs) related to photosynthesis in EC and NEC. **(B)** Chlorophyll fluorescence of F_0_ of EC and NEC. The colorful bars in the right of the pictures represent the fluorescence intensity of F_0_. White arrows pointed to ECs, black arrows denoted COR, and V-shaped arrows indicated MED. **(C)** F_v_/F_m_, qL, and Y(II) of ECs and NECs. Different letters above the bars indicated statistically significant differences (p < 0.05) between the samples by one-way ANOVA, n≥3.

Phycobilisomes (PBS) is the main light-harvesting antennae in red algae. It absorbs light energy and efficiently transfers the energy to photosystem II (PSII) and photosystem I (PS I) (Su et al., 2010). It consists of an allophycocyanin core and a lot of peripheral rods containing phycocyanin/phycoerythrin or phycoerythrin (Su et al., 2010). In this study, the coding genes of the main constituent proteins of PBS, such as the allophycocyanin linker protein coding gene, phycocyanin linker protein coding genes, and phycoerythrin linker protein coding genes, were up regulated in EC (**Figure 4A**). Therefore, these results suggested that EC could capture more light energy than NEC. Except for PBS, the coding gene of fucoxanthin chlorophyll a/c protein (FCP) was up regulated in EC. FCP could provide excellent photoprotection for algae. In diatoms, the fucoxanthin and chlorophyll bound by FCP are closely contacted, to form conjugated system coupling, which enable the excess energy absorbed by chlorophyll to be dissipated quickly and efficiently through their nearby fucoxanthin, thus the photosynthetic apparatus is protected from photodamage (Wang et al., 2019). In the present study, the up-regulation of *FCP* in EC suggested that the ability of photoprotection of EC had an advantage over NEC.

PS II is important for light reaction as it used light energy absorbed by the light-harvesting complex to split water, release oxygen, and transfer electrons to the receptor (Su et al., 2010). According to previous studies, the function of extrinsic proteins was to stabilize the activity of Mn_4_CaO_5_ cluster and optimize oxygen evolution (Enami et al., 2005). In this study, *psbU*, *psbO*, and *psbP* that encode the extrinsic proteins of PS II were up regulated in EC (**Figure 4A**). These results suggested that the PS II of EC was more active than in NEC. Moreover, the coding gene for cytochrome b6-f complex and F-ATPase were up regulated in EC. The cytochrome-b6 f complex mediates electron transport between PS II and PS I, converting the redox energy into a high-energy intermediate for ATP formation by F-ATPase (Vallon et al., 1991; Mccarty et al., 2000). These results suggested that the light reaction of EC could provide more ATP to the dark reaction for CO_2_ fixation.

Calvin cycle is a series of enzymatic reactions that assimilate CO_2_ for the primary production of organic substance in plants and algae (Frolov et al., 2019). Rubisco is a key enzyme involved in Calvin cycle, converting CO_2_ and ribulose-1,5-bisphosphate (RuBP) to six carbon compounds in red algae and in other photosynthetic organisms (Parry et al., 2012). It is sensitive to binding of its substrate RuBP or other carbon status; therefore, it could regulate photosynthesis activity effectively (Singh et al., 2022). In the present study, *Rubisco* is up regulated in EC, suggesting that carbon fixation rate of EC was higher than NEC.

Chlorophyll fluorescence could reflect the photosynthesis activity of plants (Kitajima & Butler, 1975; Genty et al., 1989; Kramer et al., 2004). To investigate the chlorophyll fluorescence of EC and NEC in the branch, microscopic chlorophyll fluorescence techniques were applied in this study. The results showed that F_0_ decreased from ECs to NECs (EC>COR>MED) (**Figure 4B**). To exclude the interference caused by cell superposition, the F_0_ of single *G. lemaneiformis* cells were measured. It was revealed that the variation in the F_0_ of single EC and NEC was consistent with that of cells in the transverse section (**Figure 4B**). Moreover, F_v_/F_m_, qL and Y(II) decreased from ECs to NECs (EC>COR>MED) (**Figure 4C**). These results suggested that EC has stronger photosynthesis than NEC, which were consistent with the results of expression of the genes related to photosynthesis.

### The genes involved in low molecular weight carbohydrates metabolism of EC and NEC

**Figure 5:**
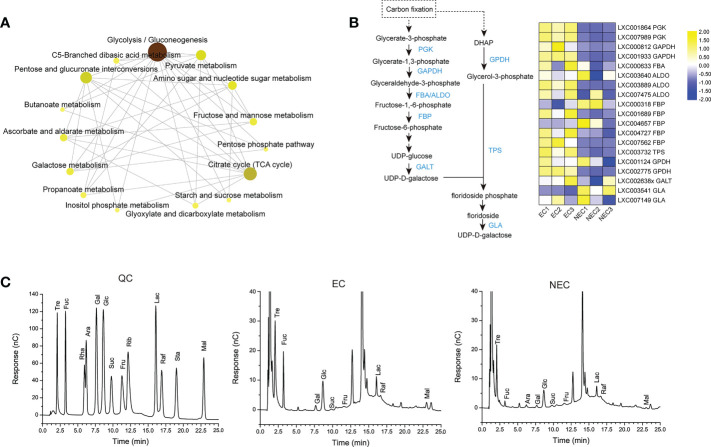
Low molecular weight carbohydrates (LMWC) metabolism in EC and NEC. **(A)** KEGG network map of 15 carbohydrate pathways enriched by differentially expressed genes (DEGs) of EC and NEC. The darker the color, the stronger the correlation between this pathway and other pathways. **(B)** Heat map displaying the expression level of some differentially expressed genes (DEGs) in glycolysis/gluconeogenesis pathway and floridoside metabolism pathway in EC and NEC. PGK: phosphoglycerate kinase, GAPDH: glyceraldehyde 3-phosphate dehydrogenase, FBA and ALDO: fructose-bisphosphate aldolase, FBP: fructose-1,6-bisphosphatase, GALT: galactose-1-phosphate uridylyltransferase, DHAP: dihydroxyacetone phosphate, GPDH: NAD-dependent glycerol-3-phosphate dehydrogenase, TPS: trehalose-6-phosphate synthase, GLA: α-galactosidase. **(C)** Chromatograms of soluble sugars in EC and NEC. X axis was the retention time and y axis was the response value. QC was the chromatogram of standard sample. Tre, Trehalose; Fuc, Fucose; Rha, Rhamnose; Ara, Arabinose; Gal, Galactose; Glc, Glucose; Suc, Sucrose; Fru, Fructose; Rib, Ribose; Lac, Lactose; Raf, Raffinose; Sta, Stachyose tetrahydrate; Mal, Maltose.

According to KEGG enrichment, the DEGs of EC and NEC were enriched in 15 pathways of carbohydrate metabolism (**Figure 5A**; **Table S3**). Among these pathways, “Glycolysis/Gluconeogenesis” interacted directly with most other pathways (**Figure 5A**), suggesting that “Glycolysis/Gluconeogenesis” played an important role in the carbohydrate metabolism in EC and NEC. Isotope labeling experiments showed that a large amount of newly-fixed carbon would flow into low molecular weight carbohydrates (LMWC) in red algae (Kroen & Ramus, 1991). According to KEGG pathway map of “Glycolysis/Gluconeogenesis”, glycrate-3-phosphate produced by photosynthesis could be converted to fructose-6-phosphate catalyzed by a series of enzymes including phosphoglycerate kinase (PGK), glyceraldehyde-3-phosphate dehydrogenase (GAPDH), fructose-bisphosphate aldolase (FAB/ALDO), and fructose-1,6-bisphosphatase (FBP). FBP is a key enzyme for gluconeogenesis which catalyzes the dephosphorylation of fructose 1,6-bisphosphate to fructose 6-phosphate (Hofius and Börnke, 2007). In the current study, *FBP*, *PGK*, *GAPDH*, and *FAB/ALDO* were up regulated in EC, which suggested that carbon fixed by photosynthesis in EC may largely flow into fructose 6-phosphate compared with NEC (**Figure 5B**). Fructose-6-P is a precursor substance of many LMWC, such as sucrose, raffinose, glucose, and floridoside (Hofius and Börnke, 2007; Lemoine et al., 2013; Yu et al., 2021). In this study, the content of 13 LMWC which related to the DEGs-enriched carbohydrate metabolism pathways, such as “Starch and sucrose metabolism” and “Galactose metabolism”, were detected in EC and NEC. The content of most of the sugars in EC were higher than in NEC (**Figure 5C**). The content of sucrose and raffinose was also found in EC and NEC, although their content had a low level.

Floridoside, one of the LMWC, is the major photosynthetic product in *G. lemaneiformis* (Broberg et al., 1998), and it is normally considered to be produced in the early stage of photosynthesis (Bondu et al., 2009). It was produced from dephosphorylated by specific phospholipase of floridoside phosphate, a product converted from glycerol-3-phosphate and UDP-D-galactose by floridoside phosphate synthase (Yu et al., 2021). Trehalose-6-phosphate synthase (TPS) was reported to have the enzymatic activity of floridoside phosphate synthase (Sun et al., 2019). In the present study, the encoding gene of TPS was up regulated in EC (**Figure 5B**). Moreover, the encoding gene of NAD-dependent glycerol-3-phosphate dehydrogenase (GPDH), an enzyme catalyzed dihydroxyacetone phosphate (DHAP) (a compound could be synthesized from early photosynthetic product) to be glycerol-3-phosphate (Yu et al., 2021), was also up regulated in ECs (**Figure 5B**). Furthermore, UDP-D-galactose could be converted from UDP-glucose and galactose-1-phosphate by galactose-1-phosphate uridylyltransferase (GALT) and the gene expression result showed that *GALT* was up regulated in EC (**Figure 5B**). This suggested that floridioside was actively synthesized in ECs of *G. lemaneiformis* under normal light condition. However, the gene of α-galactosidase (GLA), an enzyme, showed a degradation activity to floridoside in *Gracilaria tenuistipitata* and *G. sordida* (Yu & Pedersén, 1990), and was up regulated in NEC (**Figure 5B**). This suggested that the degradation activity of floridoside in NEC was higher than that in EC.

### Agar and floridean starch metabolism of EC and NEC

**Figure 6:**
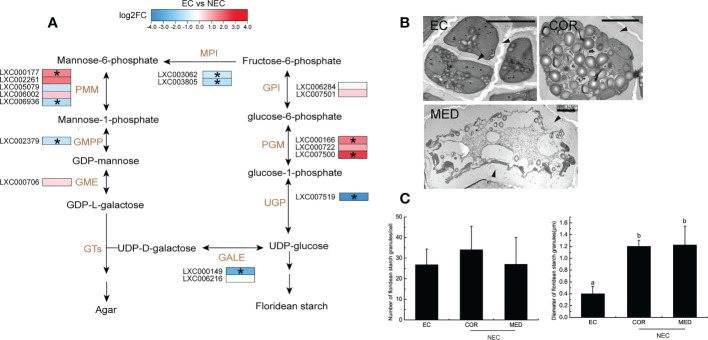
Agar and floridean starch metabolism of EC and NEC. **(A)** Heat map shows the expression level of differentially expressed genes (DEGs) in the metabolism pathway of agar and floridean starch in EC and NEC. Metabolic pathway was as described by Lee et al. (2017) and Yu et al. (2021). GPI: glucose-6-phosphate isomerase; PGM: phosphoglucomutase; UGP: UTP–glucose-1-phosphate uridylyltransferase; GALE: UDP-glucose-4-epimerase; MPI: mannose-6-phosphate isomerase; PMM: phosphomannose mutase; GMPP: GDP-mannose pyrophosphorylase; GME: GDP-mannose-3,5-epimerase, GT: glycosyltransferase. Log2FC: Log_2_(Fold Change). **(B)** Electron micrograph of cell wall and floridean starch granules of EC, COR and MED. Arrows indicated the cell wall of the cells. Scar bar=5mμ. **(C)** Number and diameter of floridean starch granules in EC and NEC. Different letters above the bars indicated statistically significant differences (p < 0.05) between the samples by one-way ANOVA, n≥3.

The agar biosynthesis begins with alternating UDP-D-galactose and GDP-L-galactose glycosylation in the Golgi apparatus (Lee et al., 2017). GDP-L- galactose could be translated from fructose-6-phosphate by many enzymes, including mannose-6-phosphate isomerase (MPI), phosphomannose mutase (PMM), GDP-mannose pyrophosphorylase (GMPP), and GDP-mannose-3,5-epimerase (GME). In this study, the *MPI*, *PMM*, and *GMPP* were significantly up regulated in NEC (**Figure 6A**), suggesting that GDP-L-galactose was synthesized more actively in NEC. UDP-D-galactose is converted from UDP-glucose and this process could be catalyzed by UDP-glucose-4-epimerase (GALE). In the current study, the GALE were significantly up regulated in NEC. The UDP-D-galactose and GDP-L-galactose in Golgi apparatus are assembled rapidly to be polysaccharide chains and then the polysaccharide is sulfated before it is transferred to the cell wall matrix (Breton et al., 2005; Lee et al., 2017). In the present study, electron microscope observations showed that the cell wall of NECs were thicker than that of EC (**Figure 6B**). These results suggested that the synthesis of agar is more active in NEC compared to EC.

Floridean starch of red algae shows structural similarities with higher plant starch but they lack amylose (Viola et al., 2001). Starch synthesis in higher plants requires the conversion of glucose-1-phosphate into ADP-glucose by AGPase as a precursor of starch synthesis. However, the synthesis of floridioside starch requires UDP-glucose rather than ADP-glucose (Dauvillée et al., 2009). UDP-glucose is produced from glucose-1-phosphate by UTP–glucose-1-phosphate uridylyltransferase (UGP). In the present study, *UGP* was significantly up regulated in NEC (**Figure 6A**), suggesting that the precursor of floridean starch was synthesized actively in NEC. Moreover, electron microscope observations showed that floridean starch content in NEC was more than that in EC (Fig 6B and 6C). These results suggested that the synthesis of floridean starch in NEC was more active than in EC.

### Expression of the genes related to photoassimilates transport routes between EC and NEC

Studies have shown that carbohydrates in apoplast could be taken up by endocytosis (Oparka & Prior, 1988). In this study, the endocytosis pathways were enriched by DEGs (**Table 3**). In mammals, the initiation module of endocytosis consists of at least the heterotetrameric adaptor protein AP2 complex and the epidermal growth factor receptor substrate 15 (EPS15), epsin and actin (Kaksonen & Roux, 2018). In the present study, the gene of EPS15(LXC003082), epsin (LXC007122), and actin (LXC000078) were up regulated in NEC. After the endocytic vesicle is detached from the plasma membrane, the substances internalized by endocytosis will be ubiquitinated and transported to the lysosome to be degraded. The endosomal sorting complexes required for transport (ESCRT) family constitutes a highly coordinated system to coordinate the sorting and enrichment of substances to be degraded for the controlled delivery of these substances to lysosomes (Cullen & Steinberg, 2018). In the present study, ESCRT-I-related genes (LXC007366 and LXC001131), ESCRT-II-related genes (LXC007049) and ESCRT-III-related genes (LXC003815 and LXC002592) were up-regulated in NEC.

**Figure 7:**
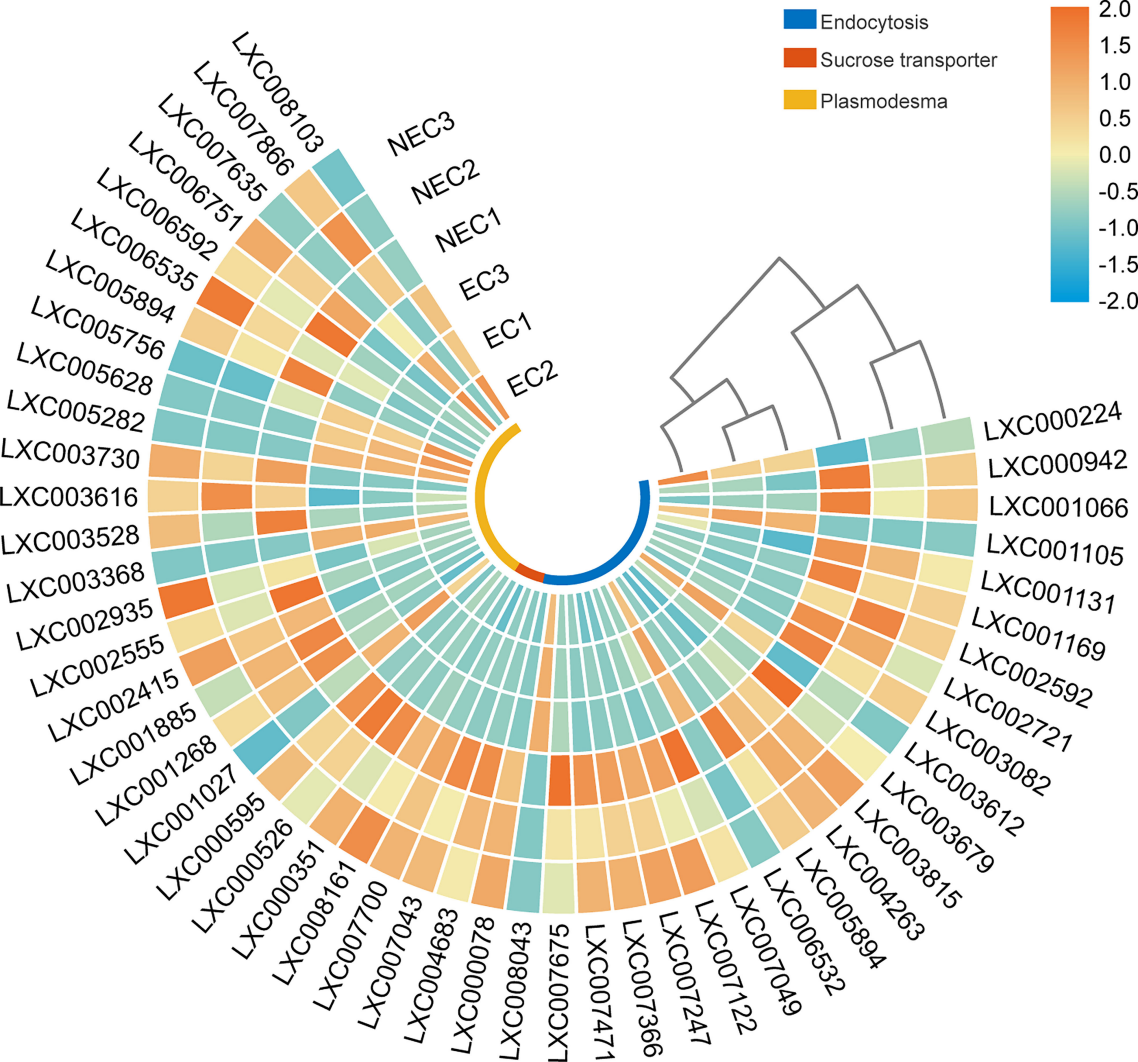
Heat map displaying the expression level of differentially expressed genes (DEGs) in photoassimilate transportation in EC and NEC.

Moreover, specific membrane transporters also play an important role in the apoplast transport pathway for photoassimilate allocation. Sucrose transporter proteins have the function of transporting sucrose across the membrane (Hofius and Börnke, 2007; Osorio et al., 2014). In this study, we found the sucrose transporter genes (LXC004683, LXC007043, LXC007700, and LXC008161) were significantly up regulated in NEC (**Figure 7**).

In addition to the apoplast route, photoassimilates could also be transported between cells *via* the symplast route. Plasmodesmata is an important symplastic transporting route for photoassimilate allocation between cells in higher plants (Hofius and Börnke, 2007). In the present study, GO terms of “plasmodesma” were enriched by DEGs of EC vs NEC and the expression of these DEGs in the cells were analyzed (**Figure 7**). Ribosomal protein RPL15 was reported to interact with the chloroplast RNA helicase and affect intercellular trafficking through plasmodesmata (Bobik et al., 2019). The encoding genes of ribosomal protein RPL13 (LXC007866), a homologue of RPL15, were up regulated in NEC. Moreover, *Kinesin* (LXC002935), which encodes a protein, localizes to microtubules and shows a function of facilitating the passage of intercellular substances through plasmodesmata (Spiegelman et al., 2018), was up regulated in NEC.

## Discussion

In the present study, single-cell transcriptome sequencing was performed to reveal the cell-type-specific in *G. lemaneiformis*. Our results from DEGs function annotation suggested that there were big differentiations in photosynthesis and metabolism between EC and NEC (**Table 2**, **Figure 3**). In most higher plants, carbon fixed in photosynthetic cells (source cell) is often converted into LMWC and then the LMWC is transported into sink cells through phloem (Ludewig & Flügge, 2013). In the current study, the photosynthesis activity and ability of EC were more than NEC. Moreover, the LMWC in EC had a higher level than in NEC (**Figures 5B, C**). This suggested that EC may function as a source cell in the strains of *G. lemaneiformis*. Agar and floridean starch are the main carbon sinks in *Gracilaia/Gracilariopsis* species and in many other Rhodophyta (Ekman et al., 1991; Viola et al., 2001). In *Gracilaria domingensis*, there was much more floridean starch in NEC than in EC (Dos Santos et al., 2013). In the current study, similar results were obtained in *G. lemaneiformis* (**Figures 6B, C**). Furthermore, a previous study has shown that the floridean starch stored in NECs of *G. lemaneiformis* were gradually decreased during the propagation of the alga (Mi et al., 2017). This implied that NECs were similar to some sink tissues/organs (storage organs like tubers) of higher plants.

Many studies have proved that photoassimilates would be transported from source cells to sink cells in higher plants (Hofius and Börnke, 2007; Lemoine et al., 2013; Osorio et al., 2014). Although there is no differentiation in vascular system, the allocation of carbon between cells has been found in algae. In *Undaria pinnatifida*, the labelled photoassimilates were translocated from the epidermis to the midrib medulla over 20min when the blade was exposed to NaH^14^CO_3_ (Wu & Meng, 1997). In the present study, many DEGs were annotated to relate to photoassimilates’ transportation (**Figure 7**), suggesting that photoassimilates produced in EC were transported to NEC to support their growth and metabolism.

In higher plants, LMWC served as transient storage pools for newly fixed carbon transported from source cells to sink cells (Hofius and Börnke, 2007; Lemoine et al., 2013; Ludewig & Flügge, 2013; Lee et al., 2020). Sucrose was the main LMWC transported between cells to keep the carbon balance of plants (Lemoine et al., 2013). Although the content of sucrose had a low level in EC and NEC, our results showed that the genes of sucrose transporters were expressed in ECs and NECs (**Figure 7**). These results suggested that sucrose might be transported between these cells. According to previous studies, floridoside in red algae showed similarities with sucrose in higher plants. First, they were the main LMWC produced in the early stage of photosynthesis. In higher plants, sucrose as a canonical transport sugar constituted 87% of ^14^C labeled photoassimilate in leaves (Yadav et al., 2021). In *G. lemaneiformis*, as well as in many red algae, floridoside is the major photosynthetic product (Broberg et al., 1998, Li et al., 2001). In *Solieria chordalis*, the photosynthetic carbon flux was responsible for 70% of the floridoside synthetized at normal conditions (Bondu et al., 2009). Second, both sucrose and floridoside could be used as a source of carbon and energy for cells. In storage organs of higher plants, carbon derived from sucrose can be stored as starch, oil, or protein (Ludewig & Flügge, 2013). Similarly, floridoside in red algae were easily interconverted to carbon sinks. After a treatment with [^14^C] bicarbonate for 48h in *Porphyridium* sp., the specific radioactivity of the floridoside fraction decreased by 80%, but the labeled soluble polysaccharide and starch were increased (Li et al., 2001). Conversely, a previous study has shown that floridean starch degradation occurred under darkness in *G. sordida*, while floridoside pool increased significantly under this condition, but the total algal carbon content was almost unchanged, suggesting that the carbon derived from floridean starch degradation could be recycled for biosynthesis diverted through floridoside (Ekman et al., 1991). In this study, gene expression results hinted that floridioside was actively synthesized in ECs while it was actively degraded in NEC (**Figure 5B**). These suggested that floridoside might act as a short-term reserve carbon pool for the transport of organic carbon in *G. lemaneiformis*.

According to previous studies, apoplast pathway is one of photoassimilate transport routes between cells (Hofius and Börnke, 2007). In the current study, many DEGs were annotated in “extracellur region” and most of these DEGs (75%) were up regulated in EC (**Figure 3**), suggesting that more substances might be transported to apoplast from EC compared to NEC. Endocytosis, a vesicle transport process that could bring nutrition into cell from extracellular space, was enriched by the DEGs (**Table 3**). Moreover, gene expression results suggested that the activity of endocytosis in NEC was more than in EC (**Figure 7**). Studies have shown that photoassimilates in apoplast could be taken up by endocytosis in storage tissue of potato tuber (Oparka & Prior, 1988). These suggested that NEC may bring the nutrients (photoassimilates) from apoplast into cells for absorption and utilization *via* endocytosis. In addition, plasmodesmata played an important role in the symplasmic transport of photoassimilates (Hofius and Börnke, 2007). In the current study, the genes that facilitate the transport of photoassimilates in plasmodesmata were expressed in EC and NEC (**Figure 7**). Pit connection is the physical connection between red algal cells, and it has been proven to transport the intercellular nutrients like the plasmodesmata of higher plants (Kim et al., 2022). In *G. lemaneiformis*, pit connection has been found to exist between EC and NEC (Mi et al., 2017). This suggested that photoassimilates of EC could be transported to NEC *via* pit connection.

**Figure 8:**
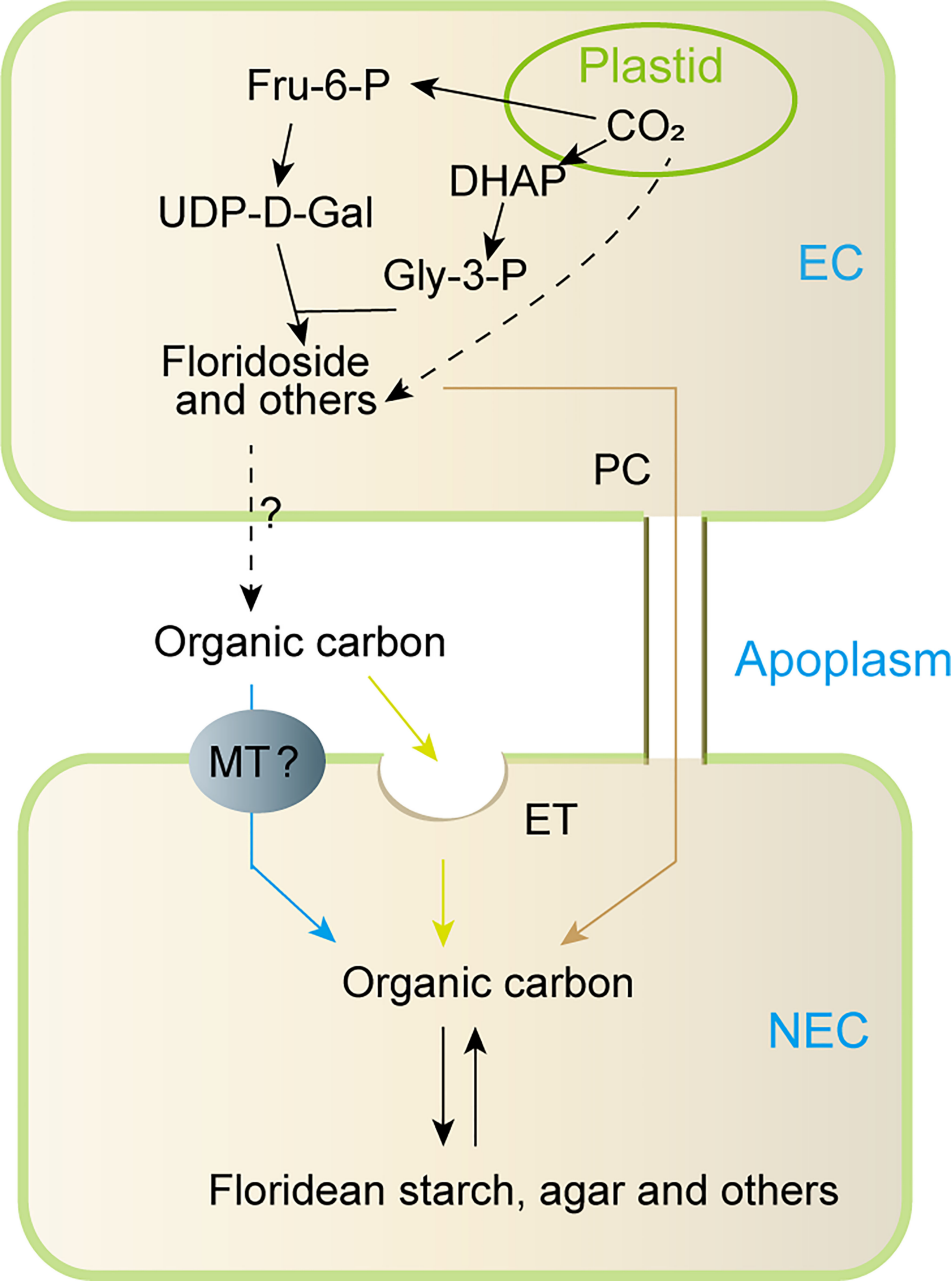
Proposed model for metabolism and transportation of organic carbon between EC and NEC of non reproductive *G. lemaneiformis* under normal light condition. Fru-6-P: fructose-6-phosphate, DHAP: dihydroxyacetone phosphate, UDP-D-Gal: UDP-D-galactose, Gly-3-P: glycerol-3-phosphate, EC: epidermal cells, NEC, non-epidermal cells; MT, membrane transporter; ET, endocytosis; PC, pit connection; “?”, uncertainty process.

Based on the previous studies and our findings, a model for carbon allocation from EC to NEC was proposed in this study (**Figure 8**). In short, the carbon fixed by photosynthesis in the EC may be converted into Fru-6-P, which then was used to synthesize LMWC (including floridoside, sucrose, and other LMWC). In addition, the carbon fixed by photosynthesis might also be used to synthesize other organic carbons (Broberg et al., 1998; Bondu et al., 2009). These organic carbons (LMWC and others) could be transported to the apoplast, in which they may be internalized into NEC through endocytosis or specific membrane transporters, and they may also be transported into NEC through pit connection. However, we currently do not know how these organic carbons were transported to apoplast from EC. In higher plants, characterized membrane transporters such as SWEETs and sucrose transporters provide transport activity for cellular efflux of sugar (such as sucrose) into the apoplast (Osorio et al., 2014; Lee et al., 2020), and cells could also use exocytosis to release intracellular organic carbons into extracellular spaces (Chin et al., 2004). This suggests that organic carbons of ECs might be transported to apoplast by specific transmembrane transporters and/or exocytosis. However, these need to be verified in future studies.

## Data availability statement

The original contributions presented in the study are publicly available. This data can be found here: NCBI, PRJNA752461.

## Author contributions

HC and ZS contributed to conception and design of the study. HC and YH organized the database and performed the statistical analysis. HC wrote the first draft of the manuscript. PL, XF, and MJ wrote sections of the manuscript. All authors contributed to the article and approved the submitted version.

## Funding

This work was supported by Shandong Provincial Natural Science Foundation (ZR2022QC090), China Agriculture Research System of MOF and MARA (CARS-50), National Natural Science Foundation of China (NO. 32072953) and Postdoctoral Applied Research Project of Qingdao (2021).

## Conflict of interest

The authors declare that the research was conducted in the absence of any commercial or financial relationships that could be construed as a potential conflict of interest.

## Publisher’s note

All claims expressed in this article are solely those of the authors and do not necessarily represent those of their affiliated organizations, or those of the publisher, the editors and the reviewers. Any product that may be evaluated in this article, or claim that may be made by its manufacturer, is not guaranteed or endorsed by the publisher.
